# Vestibular Preservation After Cochlear Implantation Using the Round Window Approach

**DOI:** 10.3389/fneur.2021.656592

**Published:** 2021-04-27

**Authors:** Keita Tsukada, Shin-ichi Usami

**Affiliations:** ^1^Department of Otolaryngology, Shinshu University School of Medicine, Matsumoto, Japan; ^2^Department of Hearing Implant Sciences, Shinshu University School of Medicine, Matsumoto, Japan

**Keywords:** cochlear implant, vestibular function, round window approach, caloric testing, cVEMP, oVEMP

## Abstract

**Background:** The development of less traumatic surgical techniques, such as the round window approach (RWA), as well as the use of flexible electrodes and post-operative steroid administration have enabled the preservation of residual hearing after cochlear implantation (CI) surgery. However, consideration must still be given to the complications that can accompany CI. One such potential complication is the impairment of vestibular function with resulting vertigo symptoms. The aim of our current study was to examine the changes in vestibular function after implantation in patients who received CI using less traumatic surgery, particularly the RWA technique.

**Methods:** Sixty-six patients who received CI in our center were examined by caloric testing, cervical vestibular evoked myogenic potential (cVEMP) and ocular VEMP (oVEMP) before or after implantation, or both, to obtain data on semicircular canal, saccular and utricular function, respectively. Less traumatic CI surgery was performed by the use of the RWA and insertion of flexible electrodes such as MED-EL FLEX soft, FLEX 28, and FLEX 24 (Innsbruck, Austria).

**Results:** Caloric response and the asymmetry ratio of cVEMP and oVEMP were examined before and after implantation using less traumatic surgical techniques. Compared with before implantation, 93.9, 82.4, and 92.5% of the patients showed preserved vestibular function after implantation based on caloric testing, cVEMP and oVEMP results, respectively. We also examined the results for vestibular function by a comparison of the 66 patients using the RWA and flexible electrodes, and 17 patients who underwent cochleostomy and insertion of conventional or hard electrodes. We measured responses using caloric testing, cVEMP and oVEMP in patients after CI. There were no differences in the frequencies of abnormal caloric and oVEMP results in the implanted ears between the RWA and cochleostomy. On the other hand, the frequency of abnormal cVEMP responses in the implanted ears in the patients who received implantation by cochleostomy was significantly higher than that in the patients undergoing surgery using the RWA.

**Conclusion:** Patients receiving CI using less traumatic surgical techniques such as RWA and flexible electrodes have reduced risk of damage to vestibular function.

## Introduction

Recently, the development of techniques such as less traumatic surgery has enabled the preservation of residual hearing and of cochlear structures after cochlear implantation (CI) surgery (1, 2). These techniques include the use of flexible electrodes (3, 4), the round window approach (RWA) (5) and steroid administration. However, consideration must still be given to the non-hearing vestibular complications that can accompany CI, resulting in balance symptoms. The incidence of vestibular symptoms, as reported previously, varies quite widely from 0.33 to 75% (6).

While there have been numerous reports evaluating the effects of CI on vestibular function, there have been few reports examining the post-operative effects of the use of a less traumatic surgical technique on vestibular function. Our previous preliminary report showed that a less traumatic technique involving the RWA is preferable from the viewpoint of vestibular preservation (7).

The aim of this study was to evaluate vestibular function before and after implantation by use of less traumatic surgical techniques, and whether such surgical techniques, particularly the RWA, result in less trauma to the vestibular end organs.

## Materials and Methods

### Patients

A total of 66 patients (21 males and 45 females) who underwent unilateral CI surgery in our center between 2009 and 2019 were included in this study after obtaining informed written consent. The 66 patients included part of the study population for whom the results of vestibular function were previously published in 2013 (8). The age at CI surgery ranged from 7 to 70 years, with a mean age of 46.6 ± 18.3 years. Twenty-five patients received implants in the right ear and forty-one in the left ear.

To preserve hearing and/or inner ear structures, we used less traumatic techniques for these patients. The less traumatic surgeries were performed by the use of flexible electrodes such as MED-EL FLEX 24™ and FLEX 28™ or FLEX soft™ electrodes (Innsbruck, Austria). The FLEX 24 electrode was implanted in 24 patients, FLEX 28 in 38, and FLEX soft in 5 patients. The full insertion of electrodes was achieved in all patients. All surgeries involving the RWA were performed by a single surgeon (S.U). With regard to steroids, systemic steroid administration was applied in patients receiving electric acoustic stimulation (EAS) using FLEX24 (2). However, steroids were not routinely used for conventional CI.

In this study, hearing thresholds, assessed by pure-tone audiometry (PTA), were measured pre- and at 6 months to 1 year post-operatively. The hearing levels were calculated by the average hearing levels (HL) at 500, 1,000, and 2,000 Hz, and the average low-frequency hearing thresholds of 125, 250, and 500 Hz (LFA) were also calculated.

The final position of the implanted electrode array was assessed by X-ray images of the horizontal plane of the cochlear basal turn obtained using the modified Stenver's view. We measured the insertion depth angel (IDA) based on the method for the determination of insertion depth described by Trieger et al. (9).

To compare surgical techniques, we also evaluated post-operative vestibular function in 17 age-matched patients (mean age: 41.6 ± 21.1, six males and eleven females) who underwent cochleostomy between 2001 and 2009. These patients had MED-EL standard™, CI24M™ or CI24R(CS)™ (Sydney, Australia) electrodes inserted.

### Vestibular Testing

The patients underwent caloric testing, cervical vestibular evoked myogenic potential (cVEMP), and ocular VEMP (oVEMP) both before or at 6 months−1 year after CI surgery, or both, to obtain data on semicircular canal function, saccular function and utricular function, respectively.

With regard to cVEMP testing, electromyography (EMG) was performed using a pair of surface electrodes mounted on the upper half and the sternal head of the sternocleidomastoid (SCM) muscle. The electrographic signal was recorded using a Neuropack evoked potential recorder (Nihon Kohden Co. Ltd., Tokyo, Japan). The method was described in detail in our previous report (8). The amplitude between the 13 ms positive peak and the 23 ms peak, and the background integrated EMG were measured, and the correction of the amplitude was calculated as follows (10):

*Corrected amplitude* = *amplitude of the averaged unrectified EMG (micro V)/background integrated EMG (micro V)*

oVEMP testing was measured by bone-conductive vibration (BCV). BCV was delivered in 4 ms tone bursts of 500 Hz vibration (rise/fall time = 1 ms and plateau time = 2 ms) by a hand-held 4810 mini-shaker (Bruel and Kjaer, Naerum, Denmark), which was placed on the forehead midline (Fz). The active electrode was located over the inferior orbital margin and a reference electrode was placed 2 cm below the active electrode. The ground electrode was placed on the chin. The patients lay in a supine position on the bed and looked up with head raised at ~30 degrees above straight-ahead during recording. The signals were amplified and bandpass filtered between 20 and 2,000 Hz. The stimulus intensity was 115 dB force level for 500 Hz, with an analysis time of 40 ms, and 50 responses were averaged for each run. For oVEMP, the amplitude was defined as the difference between the 10 ms negative peak (n10) and the 15 ms positive peak (p15).

The cVEMP and oVEMP asymmetry ratio was calculated as follows:

*asymmetry ratio (AR)* = (amplitude of CI side – amplitude of non-CI side)^*^100/(amplitude of CI side + *amplitude of non-CI side)*.

In this study, an asymmetry ratio of below −30% was defined as a decreased reaction on the CI side, that of over 30% as a decreased reaction on the non-CI side, and no reaction in amplitude bilaterally as bilaterally absent.

With regard to the caloric testing, the maximum slow phase velocity (mSPV) was measured by cold water irrigation (20°C, 5 ml, 20 s) (8) and was calculated as the percentage of canal paresis (CP%):

*CP%* = *(mSPV of CI side – mSPV of non-CI side)* ^*^ *100/(mSPV of CI side* + *mSPV of non-CI side)*.

We defined a CP% of below −25% as canal paresis (CP) on the CI side, over 25% as CP on the non-CI side, and below 10 deg/s of mSPV bilaterally as bilateral CP.

### Statistical Analysis

For all analyses, IBM SPSS version 26 for Windows software (Chicago, IL, USA) was used and the Wilcoxon signed-rank test applied when comparing differences between pre-operative and post-operative CP% for caloric testing or between pre- and post-operative AR for cVEMP and oVEMP. The Fisher's exact test was applied when comparing the frequencies of vestibular dysfunction and electrode length. The Mann-Whitney U-test was applied when comparing the frequencies of vestibular function and age, pre- and post-operative HL and LFA, and IDA. Statistical significance was set at *p* < 0.05.

## Results

### Vestibular Function Before CI Surgery

A summary of pre-operative vestibular function is shown in Table 1, and detailed data for each subject are shown in Supplementary Table 1.

**Table 1:** Summary of vestibular function before CI surgery.

	**Caloric testing**			**cVEMP**			**oVEMP**		
	**Normal**	**Abnormal**	***p*-value**	**Normal**	**Abnormal**	***p*-value**	**Normal**	**Abnormal**	***p*-value**
***n*** **(%)**	**45 (69.2)**	**20 (30.8)**		**41 (62.1)**	**25 (37.9)**		**32 (66.7)**	**16 (33.3)**	
Sex (male/female)	17/28	4/16	*p* = 0.25	13/28	8/17	*p* = 1.00	11/21	4/12	*p* = 0.74
Median age at implant	50 ± 18.4	54 ± 16.4	*p* = 0.23	45 ± 19.9	57 ± 9.7	*p* = 0.002	43 ± 21.1	55 ± 18.1	*p* = 0.19
Vestibular symptoms before CI (+/–)	7/38	10/10	*p* = 0.006	7/34	10/15	*p* = 0.048	8/24	7/16	*p* = 0.21
Pre-operative median HL (dB)	95.0 ± 9.6	91.3 ± 13.6	*p* = 0.21	91.3 ± 12.8	95.0 ± 11.7	*p* = 0.059	93.2 ± 10.6	95.0 ± 8.7	*p* = 0.37
Pre-operative median LFA (dB)	61.6 ± 26.1	71.6 ± 20.5	*p* = 0.19	55 ± 24.7	90 ± 20.8	*p* = 0.005	71.1 ± 25.1	85.0 ± 16.7	*p* = 0.28

*HL, average thresholds of 500, 1,000, 2,000 Hz; LFA, average low-frequency hearing thresholds of 125, 250, and 500 Hz*.

+, *with vestibular symptoms; –, without vestibular symptoms*.

Sixty-five patients were evaluated by caloric testing before CI surgery. Twenty of the 65 patients (30.8%) showed canal paresis (CP) on caloric testing, 5 patients had bilateral CP, 9 had CP on the CI side only, and 6 had CP on the non-CI side only. In the pre-operative cVEMP, 25 of the 66 patients (37.9%) had no response or decreased reaction bilaterally or unilaterally; 12 patients bilaterally, 8 patients on the CI-side, and five patients on the non-CI side. Sixteen of 48 patients (33.3%) who were evaluated pre-operatively by oVEMP showed absent or decreased reaction; seven patients on the CI side, two patients on the non-CI side, and the other seven patients bilaterally. With regard to vestibular symptoms before CI surgery, 7 of 45 (15.6%) patients with normal reactions and 10 of 20 (50.0%) patients with abnormal reactions on caloric testing had experienced some vestibular symptoms in the past. Seven of 41 (17.1%) patients with normal reactions and 10 of 25 (40.0%) with abnormal reactions on cVEMP, and 8 of 32 (25.0%) with normal reactions and 7 of 16 (43.8%) with abnormal reactions on oVEMP also had some vestibular symptoms before CI surgery. Although there was no significantly difference in vestibular symptoms between patients with normal and those with abnormal reactions on oVEMP (*p* = 0.21), the patients with abnormal reactions complained of significantly greater vestibular symptoms than did the patients with normal reactions on pre-operative caloric testing (*p* = 0.006) and cVEMP (*p* = 0.048). For the pre-operative caloric testing and oVEMP results, no significant differences between normal and abnormal reactions for sex, age, pre-operative mean HL, or pre-operative mean LFA were observed (Table 1). There were significant differences in the pre-operative cVEMP results between normal and abnormal reactions for age (*p* = 0.002) and pre-operative mean LFA (*p* = 0.005).

### Vestibular Preservation After CI Surgery

A summary of vestibular preservation is shown in Table 2 and detailed data for each subject are shown in Supplementary Table 1.

**Table 2:** The results of vestibular changes after CI surgery.

	**Caloric testing**			**cVEMP**			**oVEMP**		
	**No change**	**Decreased**	***p*-value**	**No change**	**Decreased**	***p*-value**	**No change**	**Decreased**	***p*-value**
***n*** **(%)**	**46 (93.9)**	**3 (6.1)**	–	**42 (82.4)**	**9 (17.6.)**	–	**37 (92.5)**	**3 (7.5)**	–
Sex (male/female)	16/30	0/3	*p* = 0.54	14/28	2/7	*p* = 1.00	11/26	2/1	*p* = 0.24
implanted side (R/L)	17/29	1/2	*p* = 1.00	11/31	5/4	*p* = 0.12	15/22	2/1	*p* = 0.57
Vestibular symptoms after CI (+/–)	8/38	1/2	*p* = 0.46	6/36	3/6	*p* = 0.19	8/29	0/3	*p* = 1.00
Median age at implant	51 ± 18.6	57 ± 20.78	*p* = 0.95	50 ± 18.7	36 ± 20.5	*p* = 0.39	52 ± 18.5	61 ± 6.7	*p* = 0.35
Pre-operative median HL (dB)	91.9 ± 13.9	100 ± 5.0	*p* = 0.094	90 ± 13.3	95 ± 13.3	*p* = 0.096	93.8 ± 10.5	87.5 ± 10.5	*p* = 0.96
Pre-operative median LFA (dB)	67.5 ± 25.3	76.6 ± 20.4	*p* = 0.57	54.2 ± 24.3	68.3 ± 26.2	*p* = 0.47	77.3 ± 24.6	90.0 ± 5.8	*p* = 0.25
Post-operative median HL (dB)	97.2 ± 9.5	105 ± 4.3	*p* = 0.62	96.9 ± 10.0	102 ± 3.8	*p* = 0.15	103.1 ± 7.4	105 ± 3.6	*p* = 0.16
Post-operative median LFA (dB)	83 ± 20.7	90 ± 9.5	*p* = 0.25	75.9 ± 20.3	86.6 ± 21.4	*p* = 0.74	90 ± 18.5	90 ± 0.0	*p* = 0.45
Electrodes (FLEX24/FLEX28 or soft)	18/28	1/2	*p* = 1.00	18/24	3/6	*p* = 0.72	9/28	0/3	*p* = 1.00
Median IDA (deg)	579.8 ± 78.7	657.9 ± 104.2	*p* = 0.36	564.1 ± 81.4	590.4 ± 95.9	*p* = 0.89	596.7 ± 82.2	603.3 ± 63.7	*p* = 0.74

*HL, average thresholds of 500, 1,000, 2,000 Hz; LFA, average low-frequency hearing thresholds of 125, 250, and 500 Hz. IDA, insertion depth angle*.

+, *with vestibular symptoms; –, without vestibular symptoms*.

All of the patients who underwent vestibular testing both before and after CI surgery had received CI by the RWA method and had had a FLEX 24, FLEX 28 or FLEX soft electrode inserted. We excluded the patients showing bilateral absent or unilateral absent reactions on the CI side before CI surgery. In this study, a post-operative CP% on caloric testing and AR on the cVEMP and oVEMP of 30% or more lower than the pre-operative results were defined as a decreased post-operative response.

**Figure 1:**
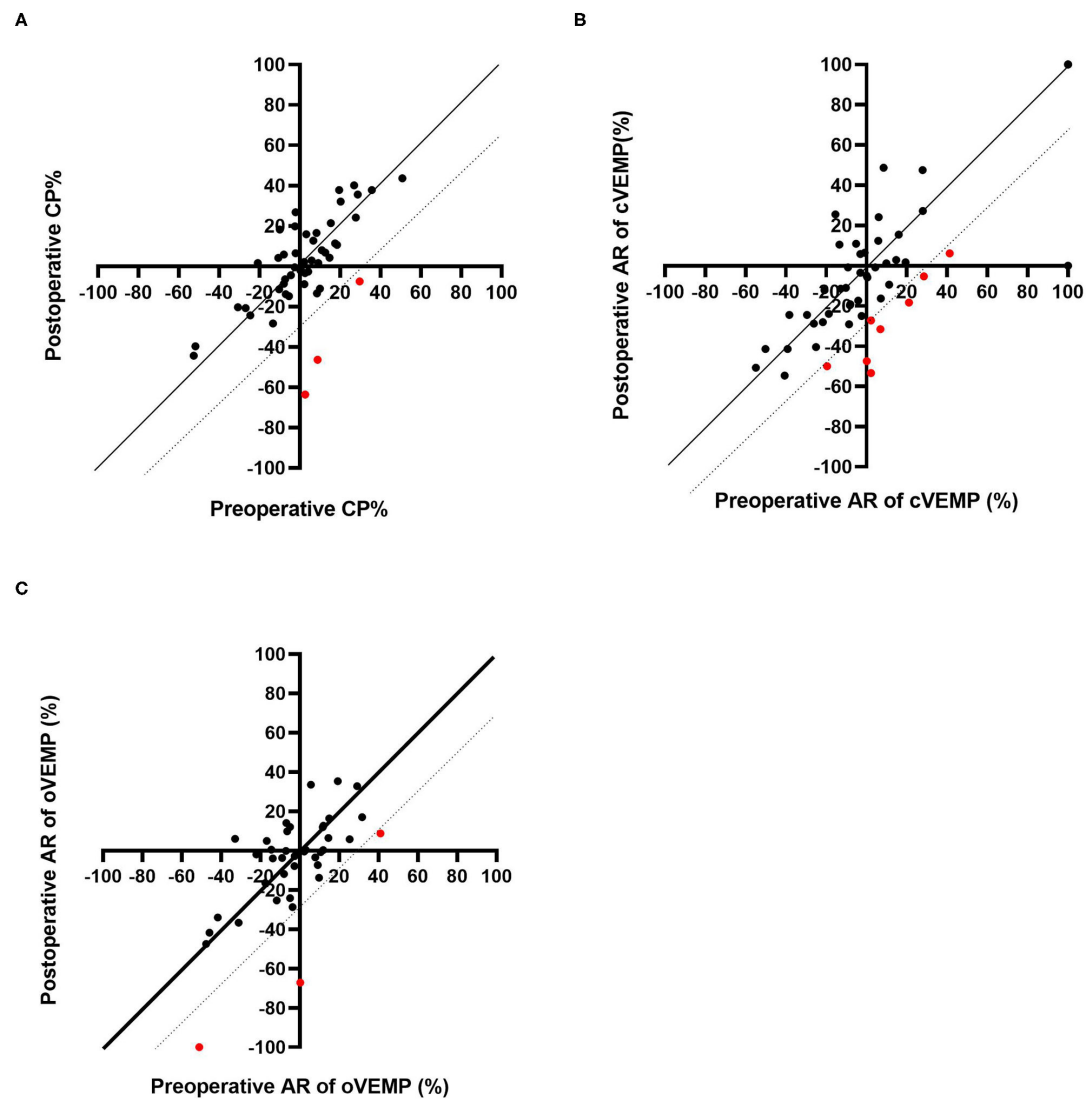
Pre-operative and post-operative CP% based on caloric testing **(A)**. Pre-operative and post-operative AR based on cVEMP **(B)** and oVEMP **(C)**. The dotted line indicates the line where the post-operative result is 30% lower than that before CI. The red dots indicate the patients who showed decreased responses post-operatively.

Caloric testing was performed before and after CI surgery in 49 patients. Figure 1A shows caloric responses before and after implantation. The pre-operative and post-operative CP% values were 2.09 ± 19.6 and 1.00 ± 23.0, respectively. There were no significant differences between caloric responses before and after implantation on caloric testing (*p* = 0.76). Compared with before implantation, the results after implantation were unchanged in 46 of 49 patients (93.9%) who underwent both pre- and post-operative testing. In the other 3 patients, the post-operative CP% was 30% or more lower than the pre-operative value.

Sixty-six patients underwent cVEMP before and after CI surgery, and we excluded 15 patients showing bilateral absent or unilateral absent reactions on the CI side before surgery. Thus, the AR values for cVEMP were compared before and after CI surgery for 51 patients (Figure 1B). The mean AR was 3.36 (SD = 34.7) pre-operatively and −4.56 (SD = 35.6) post-operatively. The post-operative AR was significantly lower than the pre-operative value on the cVEMP testing (*p* = 0.029). Forty-two of the 51 patients (82.4%) showed unchanged reactions before and after CI surgery. The other 9 patients showed decreased reactions; 8 patients showed a 30% or more reduction in AR, and the remaining patient, who had unilateral absent reaction on the non-CI side and a normal reaction on the CI side before surgery, changed to an absent reaction on the CI side, resulting in bilateral absent reaction after CI surgery.

A comparison of pre- and post-operative oVEMP results is shown in Figure 1C. Among the 48 patients who underwent oVEMP both pre- and post-operatively, we evaluated 40 patients (excluding 8 patients who had bilateral or unilateral absent reactions on the CI side before CI surgery). The pre- and post-operative AR on oVEMP was −3.60 (SD 21.5) and −6.24 (SD 26.4), respectively, and there were no significant differences observed (*p* = 0.64). Although 3 patients showed an AR reaction of 30% or more lower post-operatively when compared with the pre-operative AR, 37 of the 40 patients (92.7%) showed no change in reaction between the pre- and post-operative AR values.

Gender, implanted side, age at CI surgery, vestibular symptoms after CI surgery, and pre- and post-operative HL and LFA were compared between patients with and without preservation of vestibular function, but there were no significant differences in the results for caloric testing, cVEMP or oVEMP (Table 2). We compared results between those fitted with a shorter electrode (FLEX 24:24 mm) and those fitted with longer electrodes (FLEX 28:28 mm or FLEX soft:31.5 mm), but again no significant differences were observed between them on caloric testing (*p* = 1.00), cVEMP (*p* = 0.72) or oVEMP (*p* = 1.00). IDA was also compared between patients with and without vestibular preservation. Median IDA was 579.8 ± 78.7 deg in patients with vestibular preservation on caloric testing and 657.9 ± 104.2 deg in patients without vestibular preservation. Further, median IDA was 564.1 ± 81.4 deg and 590.4 ± 95.2 deg in patients with and without vestibular preservation on cVEMP, and 596.7 ± 82.2 deg and 603.3 ± 63.7 deg in patients with and without vestibular preservation on oVEMP, respectively (Table 2). There was no difference in IDA between the two groups on caloric testing (*p* = 0.36), cVEMP (*p* = 0.89) or oVEMP (*p* = 0.74).

### A Comparison Between RWA and Cochleostomy

As we could not evaluate pre-operative vestibular function in patients who underwent cochleostomy, we compared the post-operative vestibular function in 66 patients (mean age: 46.6 ± 18.3) who underwent CI with the RWA and 17 age-matched patients (mean age: 41.6 ± 21.1) who underwent cochleostomy. The detailed results for patients receiving cochleostomy are shown in Supplementary Table 2.

**Figure 2:**
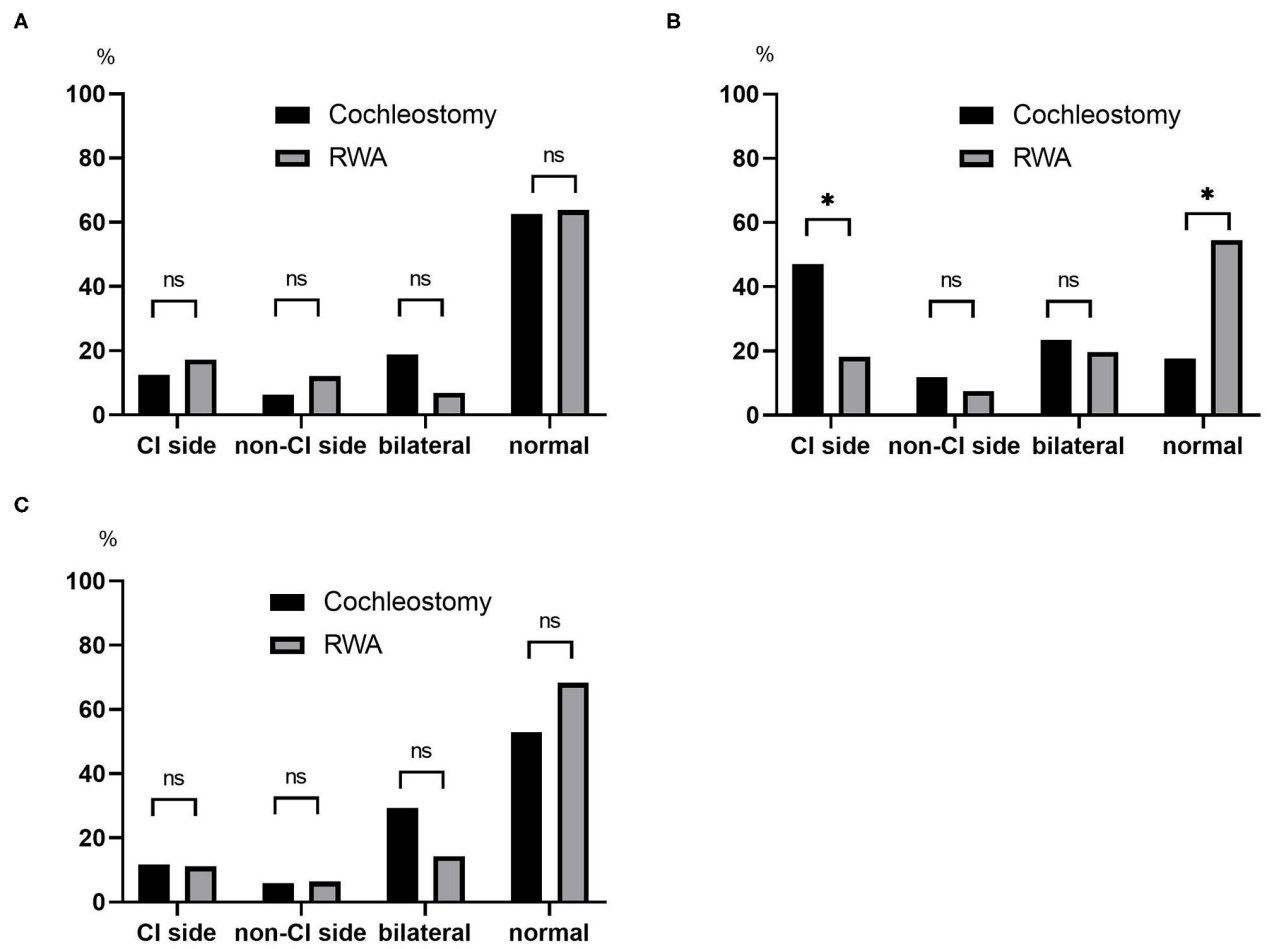
The frequencies of post-operative abnormal results on the CI side, non-CI side, bilaterally absent and post-operative normal reactions based on **(A)** caloric testing, **(B)** cVEMP, **(C)** oVEMP were compared between the RWA and cochleostomy. The frequency of abnormal post-operative cVEMP responses on the CI side in cochleostomy patients was significantly higher than that in RWA patients (*p* = 0.023). ns, not significant; *p < 0.05.

The post-operative results for vestibular function are shown in Figure 2. The frequencies of abnormal reactions on post-operative caloric testing on the CI side were 12.5% in the cochleostomy patients and 17.2% in the RWA patients (Figure 2A), and those on post-operative oVEMP were 11.8% in the cochleostomy patients and 11.1% in the RWA patients (Figure 2C). There were no significant differences in the frequencies of post-operative abnormal reactions on the CI side (*p* = 1.00 on caloric testing and oVEMP), the non-CI side (*p* = 0.689 for caloric testing, *p* = 1.00 for oVEMP), or bilaterally (*p* = 0.061, *p* = 0.162), or of bilateral normal reactions (*p* = 0.577, *p* = 0.263) between RWA patients and cochleostomy patients. On the other hand, the frequencies of decreased or absent cVEMP responses on the CI side in the RWA patients and cochleostomy cases were 18 and 47%, respectively. The frequency of abnormal post-operative cVEMP responses on the CI side in cochleostomy patients was significantly higher than that in RWA cases (*p* = 0.023), even though there were no differences in the frequency of decreased cVEMP responses on the non-CI side (*p* = 0.63) or of bilateral abnormal responses (*p* = 0.74) between the cochleostomy patients and RWA patients (Figure 2B).

## Discussion

Previous reports showed that the frequency of normal pre-operative vestibular function in patients who were candidates for CI was about 41–84% based on caloric testing (11–20), 35–89% based on cVEMP (12, 14–17, 19–23), and 50–71% based on oVEMP (19, 20, 23). In the present study, we found that the pre-operative frequencies of normal vestibular function in patients who received CI were 69.2, 62.1, and 66.7% for caloric testing (semicircular function), cVEMP (saccular function) and oVEMP (utricular function), respectively. Although the frequencies of normal responses showed some variations from those in previous studies, similar results were obtained in this study. We previously reported that patients who were candidates for EAS had relatively good vestibular function before CI surgery (8). We also evaluated the relationship between residual hearing at low frequencies and vestibular function. Although no significant differences were shown on caloric testing or oVEMP, it was found that the greater the residual hearing, the more saccular function is preserved based on pre-operative cVEMP results. Thus, we have to pay attention to the preservation of vestibular function, particularly in patients with residual hearing.

In this study, to preserve vestibular function, less traumatic CI surgical techniques (the RWA with a flexible thin electrode) were performed, and the frequencies of post-operative preservation of semicircular function, saccular function and utricular function were 93.9, 82.4, and 92.5%, respectively.

Further, in this study, we are able to perform comprehensive vestibular testing (including semicircular function, saccular function and utricular function). There have been few previous reports to date on comprehensive vestibular function. Chen et al. reported that among the vestibular functions, the semicircular canal function was more frequently damaged (19). Sonsa et al. showed that the frequencies of post-operative damage to these three vestibular functions were almost equal (20). Our present study showed that saccular function was more frequently damaged post-operatively. A previous histopathological study also showed that the saccule was the most frequently damaged organ, followed by the utricle and semicircular canals (24). The saccule is anatomically and embryologically closer to the cochlea than is the utricle or semicircular canals (25). Cytologically, dark cells, which secrete potassium and create homeostasis in the endolymph, are present in the utricle and semicircular canal ampulla, but not in the saccule (26). It is proposed that saccular endolymph originates from the cochlea by longitudinal flow or diffusion and is not produced in this organ. Based on these anatomical, embryological, and cytological aspects, it is speculated that the saccule is more susceptible to environmental changes in the cochlea due to CI surgery than are the utricle and semicircular canals.

The previously reported frequencies of post-operative preservation of vestibular functions are shown in Table 3. Post-operative preservation was found in 7–94% based on caloric testing, 0–88% based on cVEMP, and 13–95% based on oVEMP. Our results showed relatively better preservation of post-operative vestibular functions compared with those of previous reports. When considering factors related to vestibular preservation, there were no significant differences between vestibular preservation for age at implant, implanted side, gender, and pre- and post-operative HL and LFA in the present study.

**Table 3:** The frequencies of post-operative vestibular preservation in the literature.

**Preservation rate % (*****n*****)**			**Surgical approach**	**References**
**Semicircular canal function**	**Saccular function**	**Utricular function**		
66% (24)			Unknown	(27)
44% (66)			Unknown	(28)
77% (60)			Unknown	(29)
62% (8)			Cochleostomy	(11)
43% (14)			Cochleostomy	(30)
	0% (12)		Cochleostomy	(31)
68% (86)			cochleostomy	(32)
57% (21)	50% (16)		Cochleostomy	(12)
91% (27)	87% (23)		RWA	(12)
	0% (18)		Cochleostomy	(33)
42% (12)	50% (8)		Cochleostomy	(21)
68% (38)			Cochleostomy	(13)
	18% (17)		Cochleostomy	(22)
61% (89)	49% (89)		Cochleostomy	(34)
94% (16)	69% (16)		RWA	(14)
50% (16)	14% (14)		Cochleostomy	(16)
94% (17)	77% (11)		RWA	(15)
40% (20)	40% (20)		Unknown	(35)
	39% (26)	13% (22)	Cochleostomy	(23)
27% (30)	38% (29)		RWA	(36)
28% (22)	41% (22)		RWA	(17)
82% (17)	24% (17)		RWA	(18)
7% (29)	58% (22)	63% (19)	RWA	(19)
62% (21)	54% (26)		RWA	(37)
80% (10)	42% (12)		RWA	(38)
86% (10)	56% (9)		Cochleostomy	(38)
84% (55)	84% (55)	81% (55)	RWA	(20)
	88% (42)	95% (42)	RWA	(39)
79% (120)			RWA	(40)
89% (49)	84% (51)	91% (40)	RWA	This study

Thus, one of the reasons for such better outcomes is probably the surgical technique applied, such as the RWA and the use of flexible electrodes.

**Figure 3:**
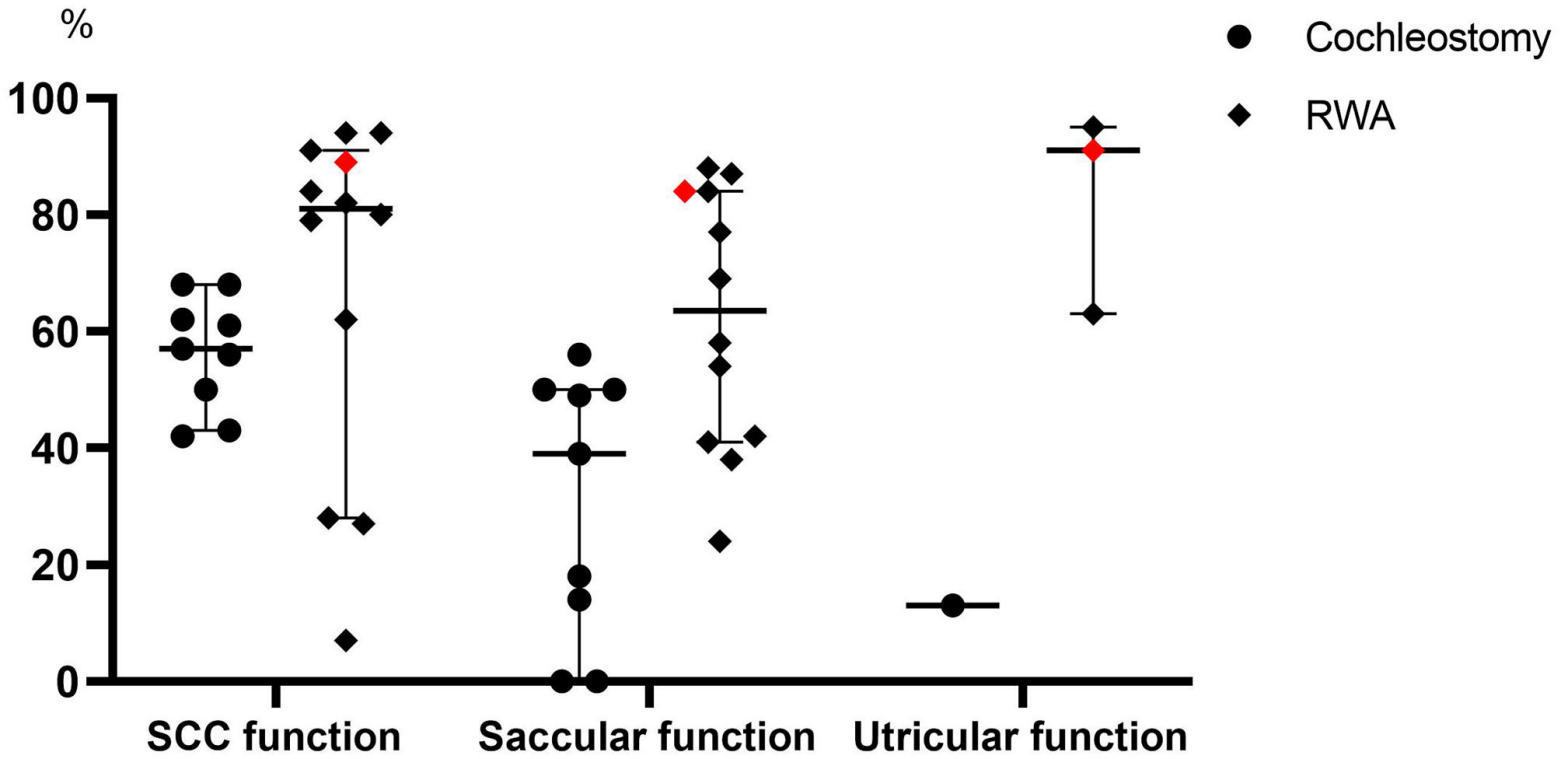
A comparison of the frequencies of vestibular preservation between patients undergoing the RWA and cochleostomy in the literature. The red dots indicate the present results. SCC, Semicircular canal.

In our present study, the frequencies of post-operative abnormal reactions on caloric testing and oVEMP in the implanted ears did not significantly differ regardless of whether the patients received the RWA or cochleostomy. On the other hand, the frequency of abnormal post-operative cVEMP results in the implanted ears was significantly higher in the cochleostomy patients than in the RWA patients. Todt et al. reported that decreased function based on post-operative cVEMP was seen in 50% of patients who received cochleostomy and in 13% of those receiving the RWA, while abnormal post-operative caloric testing results were seen in 42.9 and 9.4% of cochleostomy and RWA patients, respectively (12). We also confirmed that the RWA is a preferable approach for the preservation of vestibular function (7, 8). We compared the frequencies of vestibular preservation between RWA and cochleostomy patients in the literature (Figure 3) (11–23, 27–40). The median preservation rates for RWA and cochleostomy patients were 81 and 61% based on caloric testing, and 63.5 and 39% based on cVEMP, respectively. Although there were few oVEMP results available, the preservation rates for RWA patients were better than those for cochleostomy patients in terms of vestibular function. These results suggested that the RWA results, including those from our study, indicated less trauma to post-operative vestibular function than did those for cochleostomy, particularly in terms of saccular function.

Ishiyama previously reported that a histopathological study showed secondary fibrosis and endolymphatic hydrops when the cochleostomy involves the scala vestibuli (SV) (41). Other temporal bone studies have also shown that electrode insertion into the SV involves damage to the vestibular receptors. However, when the electrode was inserted into the scala tympani (ST), no vestibular damage was found (24). When considering the results of histopathological studies of cochleostomy and the RWA, Ishiyama reported that cochleostomy was significantly associated with SV fibrosis and hydrops whereas round window insertion was not (41). Adunka evaluated CI electrode insertions through the round window membrane histologically and reported that smooth implantations via the round window membrane resulted in deep, atraumatic insertions into the ST, and unintentional lesions to the basilar membrane could be avoided by using the round window as an exact anatomical landmark that is always in direct continuity with the ST (42). These histological studies imply that one of the reasons for our worse results on cVEMP testing among cochleostomy cases may be the dislocation of electrodes.

Thus, the histopathological and clinical results in previous studies have shown that the RWA preserves vestibular functions to the greatest extent and, therefore, is superior to cochleostomy.

In our study, all the patients who underwent RWA had flexible electrodes such as FLEX soft, FLEX28, and FLEX24 inserted, whereas the patients who underwent cochleostomy mainly had conventional or hard, peri-modiolar electrodes [MED-EL standard, CI24M and CI24R (CS)] inserted. In most of the previous studies of vestibular preservation in CI surgery, the type of electrode used varied within each study to include hard electrodes, whereas our preservation study used only flexible electrodes. A previous review of electrode designs reported that hard, peri-modiolar electrodes had a higher incidence of translocation from the ST to the SV, and also showed that the insertion force of the electrodes was lower for flexible electrodes (35–55 mN) than for other electrodes (over 75 mN) (43). Histological study and dissection of human temporal bones performed by Adunka et al. (3) confirmed the atraumatic nature of flexible electrodes. Insertion forces with the conventional standard array and FLEX array were measured in an acrylic model of the ST, demonstrating that the insertion force could be reduced significantly by more than 40% with the FLEX electrode (3). This indicates that the flexible electrodes result in less trauma to the structure of the cochlea. One of the reasons of our better preservation results using the RWA than cochleostomy in the present study and other previous reports may be that the use of such less traumatic flexible electrodes reduces trauma not only to the cochlear but also to the vestibular receptors. Thus, the use of both RWA and a flexible electrode reduced the risk of damage to vestibular function.

In this study, we also evaluated the relationship between vestibular symptoms and vestibular function. Forty to 50% of the patients who complained of some vestibular symptoms before CI surgery had vestibular dysfunction pre-operatively. They had a significantly higher frequency of vestibular dysfunction compared to patients who had not complained of vestibular symptoms. However, there were no differences in post-operative vestibular symptoms between patients with and without vestibular preservation. Therefore, vestibular symptoms are not due to operative trauma, but are due to the pre-operative pathology of the patients.

Regarding the electrode length and IDA, the preservation rates of post-operative semicircular canal function were 95 and 90% in the patients receiving FLEX 24 and FELX 28/FLEX soft electrodes, respectively. Meanwhile, 86% of patients receiving a FLEX 24 and 80% with a FLEX 28/soft, and 100% receiving a FLEX24 and 90% with a FLEX 28/soft showed preserved post-operative saccular or utricular functions, respectively, based on cVEMP and oVEMP results. We also evaluated the relationship between IDA and vestibular preservation. There were no significant differences in IDA between patients with and without vestibular preservation based on caloric testing, cVEMP and oVEMP. In our previous study (44), we reported that 17 of 18 (94.4%) patients who had residual hearing in the low frequencies retained low-frequency hearing when implanted with longer electrodes such as FLEX 28 and FLEX soft electrodes. There were no significant differences between the shorter and longer electrodes in these patients. Similarly, the cochlear function in the present results indicates that vestibular functions can be preserved even when applying longer electrodes or with deeper insertion using flexible electrodes. A previous report by Nordfald also showed that there was no significant differences in the residual hearing and vestibular function between the FLEX 28 and the FLEX soft electrodes based on caloric testing, cVEMP and SVH/SVV results (37). These results indicate that it is possible to preserve not only residual hearing but also vestibular function by use of a longer electrode. Although it cannot be excluded that cochleosotomy insertion using atraumatic electrodes will not produce the same result, preservation of vestibular function is not influenced by electrode length, but is thought to be due to the surgical technique used, such the RWA, and the use of flexible electrodes.

## Conclusion

The above results indicate that less traumatic surgical techniques such as RWA and flexible electrodes can reduce the risk of damage to vestibular function. It is important to preserve not only hearing but also vestibular function by using such techniques. Further, there were no significant differences in the frequencies of vestibular dysfunction in terms of the length of the flexible electrodes used.

## Data Availability Statement

The raw data supporting the conclusions of this article will be made available by the authors, without undue reservation.

## Ethics Statement

The studies involving human participants were reviewed and approved by Shinshu University Ethical Committee. Written informed consent to participate in this study was provided by the participants' legal guardian/next of kin.

## Author Contributions

KT designed the study, collected the data, performed data analysis, wrote the manuscript, created the figures, and performed the literature search. S-iU designed the study and revised the manuscript. All authors contributed to the article and approved the submitted version.

## Conflict of Interest

The authors declare that the research was conducted in the absence of any commercial or financial relationships that could be construed as a potential conflict of interest.
